# Magnetic sphincter augmentation versus fundoplication for GERD: a systematic review and meta-analysis of postoperative outcomes

**DOI:** 10.1097/MS9.0000000000004522

**Published:** 2026-01-28

**Authors:** Mohammed Amer Kamel, Yonatan Abbawa Zewdie, Sai Venkata Siddhartha Masetti, Mira Odeessa Pereira, Maham Afzal, Akash Rawat, Sahibzada Zumeran Jah Jah, Prachi P. Salunke, Suhas Kataveni, Zainab Shaheen, Abdulrahman Bukamal, Shahad Abu Ahmad, Mirza Muhammad Hadeed Khawar, Asraf Hussain

**Affiliations:** a Department of Medicine, Al-Quds University, Jerusalem, Palestine; b Department of Medicine, Addis Ababa University, College of Health Sciences, School of Medicine, Addis Ababa, Ethiopia; c Department of Medicine, Kingston Public Hospital, Kingston, Jamaica; d Department of Medicine, Medical University of Warsaw, Warsaw, Poland; e Department of Medicine, Shalamar Medical and Dental College, Lahore, Punjab, Pakistan; f Department of Medicine, Himalayan Institute of Medical Sciences, Swami Rama Himalayan University, Dehradun, Uttarakhand, India; g Department of Medicine, Services Institute of Medical Sciences, Lahore, Punjab, Pakistan; h Department of Medicine, Tver State Medical University, Tver, Russia; i Department of Medicine, Gandhi Medical College and Hospital, Secunderabad, Telangana, India; j Department of Medicine, Aligarh Muslim University, Aligarh, Uttar Pradesh, India; k Department of Medicine, Royal College of Surgeons in Ireland, Bahrain, Manama, Bahrain; l Department of Medicine, Chitwan Medical College, Bharatpur, Chitwan, Nepal

**Keywords:** fundoplication, gastroesophageal reflux disorder, GERD, magnetic sphincter augmentation

## Abstract

**Background:**

Gastroesophageal reflux disease (GERD) is commonly treated with surgical interventions such as magnetic sphincter augmentation (MSA) or fundoplication (FP). This meta-analysis evaluates postoperative outcomes of MSA versus FP in GERD management.

**Methods:**

A systematic literature search was conducted across PubMed, Cochrane Library, ScienceDirect, and Embase up to April 2025, identifying studies comparing MSA and FP for GERD. Pooled odds ratios (ORs) or mean differences (MDs) with 95% confidence intervals (CIs) were calculated using a random-effects model. Heterogeneity was assessed with the *I*^2^ statistic, and statistical significance was set at *P* < 0.05.

**Results:**

Twelve studies, published between 2014 and 2024, were included. No significant differences were observed in postoperative proton pump inhibitors (PPI) use (OR: 0.70, 95% CI: 0.30–1.67, *P* = 0.40, *I*^2^ = 85%) or GERD-HRQL scores (MD: 0.67, 95% CI: –0.57–1.91, *P* = 0.29, *I*^2^ = 68%) and dysphagia (OR: 1.12, 95% CI: 0.59–2.14, *P* = 0.72, *I*^2^ = 72%) between MSA and FP. MSA significantly improved the ability to belch (OR: 6.78, 95% CI: 4.49–10.22, *P* < 0.00001, *I*^2^ = 43%) and ability to vomit (OR: 5.85, 95% CI: 2.39–14.33, *P* = 0.0001, *I*^2^ = 82%) and reduced gas bloating risk (OR: 0.43, 95% CI: 0.25–0.75, *P* = 0.003, *I*^2^ = 54%) compared to FP.

**Conclusions:**

MSA and FP demonstrate comparable efficacy in GERD symptom control, PPI use, and dysphagia. MSA offers significant advantages in preserving the ability to belch and vomit and reducing gas bloating. Substantial heterogeneity in GERD-HRQL scores, postoperative PPI use, dysphagia, and ability to vomit warrants cautious interpretation, and further studies are needed to optimize surgical approaches.

## Introduction

Gastroesophageal reflux disease (GERD) is a chronic condition where stomach contents reflux into the esophagus, causing mucosal injury^[1]^. It affects about 14% of the global population^[2]^, with heartburn and regurgitation as classic symptoms, though atypical manifestations occur^[3]^. GERD can lead to serious complications like peptic strictures, Barrett’s esophagus, and esophageal carcinoma. Proton pump inhibitors (PPI) therapy and lifestyle modifications are considered the first-line management for non-refractory cases^[4]^. However, up to 50% of patients are intolerant of medical therapy and present with refractory symptoms^[5]^. These patients, along with those with complicated diseases, e.g., large hiatal hernias, are often referred to surgical interventions^[6]^.

Standard surgical techniques like laparoscopic Nissen fundoplication (FP) and more recent procedures like magnetic sphincter augmentation (MSA) using the LINX^®^ device are used to treat GERD. Laparoscopic Nissen Fundoplication (LNF), which involves wrapping the stomach fundus 360 degrees around the lower esophageal sphincter (LES) to strengthen the anti-reflux barrier^[7]^, is still a commonly used and proven treatment. Nevertheless, MSA has become a less intrusive method that augments the LES with a ring of magnetic beads, augmenting LES pressure via magnetic force to prevent reflux while allowing transient sphincter opening for belching and vomiting, thereby maintaining these physiological processes^[8]^.

Every strategy offers distinct advantages. FP has a well-established long-term success rate and is very successful at controlling symptoms^[7]^. In contrast, MSA provides quicker recuperation, shorter recovery periods, and improved preservation of functions like vomiting and belching, which are frequently compromised following FP^[8]^. Furthermore, research indicates that MSA improves GERD-related quality of life scores on par with FP^[9]^, but it also has greater rates of PPI discontinuation – up to 85.4% after the procedure^[10]^.

Both methods have disadvantages despite these advantages. Gas-bloat syndrome, dysphagia, and the inability to vomit or belch are postoperative side effects linked to FP, but MSA may have a slightly greater transitory incidence of dysphagia and device-related complications such as erosion (0.24%) and explantation (3.9%)^[10]^. Additionally, whereas MSA typically results in superior belching ability and fewer gas-bloat symptoms than LNF (95.2 vs. 65.9%, respectively), it might not be appropriate for individuals with Barrett’s esophagus or significant hiatal hernias^[8,11]^.

When choosing a patient, these elements are especially crucial. FP may still be the better choice in groups where physical anomalies such as large Hiatal hernias are common, but MSA is best for younger individuals who are drug intolerant and have no complicated esophageal pathology. As GERD surgical treatment advances, individualized treatment planning based on patient anatomy, symptoms, and preferences is still essential^[11]^.

Existing studies report variable results, with some suggesting that MSA offers comparable symptom control with fewer side effects, while others favor FP for durability. These inconsistencies, coupled with limited head-to-head trials and small sample sizes, underscore the need for a comprehensive synthesis of available evidence to guide clinical practice and optimize patient outcomes. This meta-analysis aims to compare the efficacy, safety, and long-term outcomes of MSA versus FP in adult patients with GERD, focusing on symptom relief, complication rates, and quality of life. This study adheres to the TITAN guidelines for transparency in reporting artificial intelligence (AI)^[12]^. No AI tools were used in the preparation of this manuscript.

## Methods

This systematic review and meta-analysis adhered to the Preferred Reporting Items for Systematic Reviews and Meta-Analyses (PRISMA) statement and Cochrane Collaboration guidelines^[13,14]^ (PROSPERO ID: CRD42024564433). Institutional Review Board approval was not required because the data were publicly available from electronic databases.

This systematic review and meta-analysis was conducted following a structured step-by-step procedure: (1) Defining the research question using the PICO framework: Population (adults with GERD), Intervention (MSA), Comparison (FP), and Outcomes (postoperative clinical outcomes such as GERD-HRQL scores, PPI use, dysphagia, ability to belch and vomit, and gas bloating). (2) Developing a comprehensive search strategy with keywords and Medical Subject Headings (MeSH) terms. (3) Performing the literature search in multiple databases from inception to April 2025. (4) Screening titles and abstracts independently by two reviewers, followed by full-text assessment for eligibility. (5) Extracting data from included studies using a standardized form. (6) Assessing the quality of studies with the Newcastle-Ottawa Scale (NOS). (7) Conducting statistical analyses, including meta-analysis with appropriate models, heterogeneity assessment, sensitivity analysis, publication bias evaluation, and Grading of Recommendations Assessment, Development, and Evaluation assessment for evidence certainty. (8) Interpreting the results, integrating findings with existing literature in the discussion, and drafting the manuscript with input from all authors, including revisions for clarity, consistency, and adherence to guidelines, culminating in the final version.

**HIGHLIGHTS**

- Meta-analysis compares magnetic sphincter augmentation (MSA) and fundoplication (FP) for gastroesophageal reflux disease (GERD) management.
- MSA and FP show similar efficacy in GERD symptom control, proton pump inhibitors use, and dysphagia rates.
- MSA significantly improves ability to belch (or: 6.78, *P* < 0.00001) and vomit (or: 5.85, *P* = 0.0001).
- MSA reduces gas bloating risk compared to FP (or: 0.43, *P* = 0.003).
- High heterogeneity in some outcomes suggests need for further research.

### Search string

A comprehensive literature search was conducted through PubMed, Cochrane Library, ScienceDirect, and Embase from their inception to April 2025. Reference lists of eligible articles were screened for additional relevant studies.To maximize the retrieval of relevant studies, we used a combination of keywords and MeSH terms, including “gastroesophageal reflux” OR “GERD” AND “magnetic sphincter augmentation” OR “LINX” AND “fundoplication” OR “Nissen fundoplication” OR “Toupet fundoplication” AND “postoperative outcomes” OR “complications” OR “dysphagia.”

### Eligibility criteria

**Inclusion Criteria**: Studies were included if they followed the PICO framework and involved adults (≥18 years) with GERD, compared MSA to Nissen or Toupet FP, reported postoperative outcomes, and were designed as observational studies.**Exclusion Criteria**: Studies involving pediatric populations, non-English publications, conference abstracts, editorials, or letters to the editor were excluded.

### Study selection

Two reviewers independently screened titles and abstracts using Zotero. Full texts of potentially eligible studies were retrieved and assessed against inclusion criteria. Disagreements were resolved through discussion or consultation with a third reviewer.

### Data extraction and collection

Two authors independently extracted data from selected studies using a standardized Google Sheet. Extracted data included study characteristics (e.g., author’s name, year of publication, country, and sample size), baseline characteristics (e.g., patient demographics and comorbidities), and postoperative outcomes. For postoperative PPI use, data were extracted as the proportion of patients continuing PPI therapy. In cases where studies reported PPI discontinuation rates, these were inverted to calculate PPI use (i.e., PPI use = 1 – discontinuation rate) to ensure comparability and consistency across studies. Disagreements were resolved by consensus.

### Quality assessment

The NOS was used to evaluate study quality^[15]^. Two independent authors assessed quality based on three factors: patient selection (four points), group comparability (two points), and exposure ascertainment (three points). Total scores ranged from 0 (worst) to 9 (best), with quality interpreted as good (≥7), moderate (5–6), or poor (≤4). Discrepancies were resolved by consensus.

### Statistical analysis

Data were analyzed using RevMan 5.4 (The Cochrane Collaboration, Copenhagen, Denmark)^[14]^. Continuous outcomes were evaluated using mean differences (MDs), and dichotomous outcomes were assessed using odds ratios (ORs) with 95% confidence intervals (CIs). The 95% CIs measured the precision and reliability of effect sizes, with narrower intervals indicating greater precision and wider intervals suggesting more uncertainty.Heterogeneity was assessed using the *I*^2^ statistic, guided by the Cochrane Handbook for Systematic Reviews of Interventions. A random-effects model was applied if heterogeneity was substantial (*I*^2^ > 50%) to account for variability among studies^[16]^; otherwise, a fixed-effect model was used. For significant heterogeneity, a leave-one-out sensitivity analysis was conducted to evaluate the influence of individual studies. Forest plots were generated to visualize study-level and pooled estimates. Publication bias was assessed using funnel plots. Following the statistical analysis, the quality of evidence for each outcome was evaluated using the Grading of Recommendations Assessment, Development, and Evaluation (GRADE) approach, assessing domains such as risk of bias, inconsistency, imprecision, indirectness, and effect size to determine the certainty of evidence^[17]^.

#### Model selection per outcome

The choice of meta-analytic model was determined for each outcome based on the *I*^2^ statistic threshold: a fixed-effects model was employed if *I*^2^ ≤ 50% (indicating low to moderate heterogeneity), while a random-effects model was used if *I*^2^ > 50% (indicating substantial heterogeneity). This approach ensured appropriate accounting for between-study variability. The specific model applied to each outcome is detailed in the results section.

## Results

### Study selection

**Figure 1.**
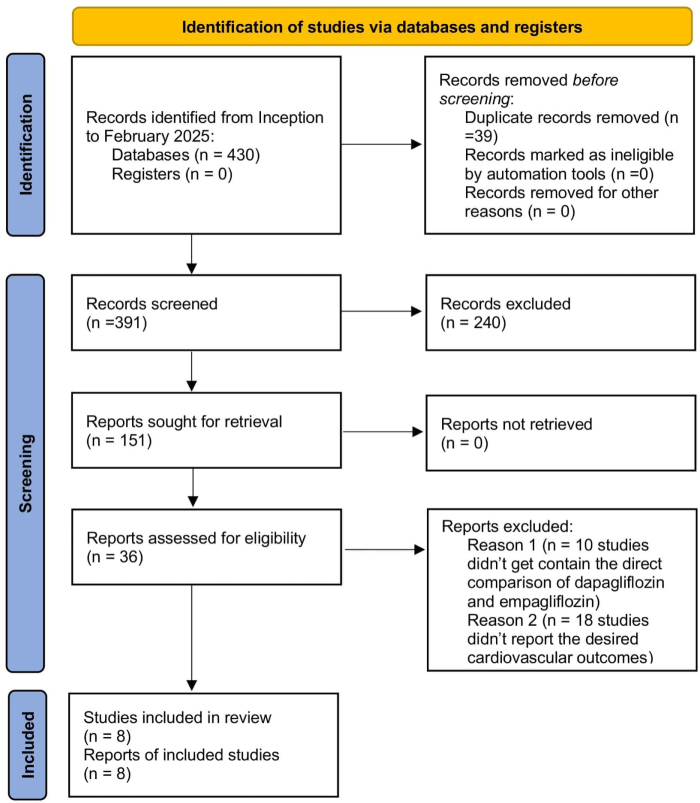
Preferred Reporting Items for Systematic Reviews and Meta-Analyses flowchart outlining the literature screening process, study selection, and exclusion criteria.

The PRISMA statement flowchart (Fig. 1) outlines the literature screening process and study selection. The initial search yielded 589 articles, from which 36 full-text articles were retrieved for assessment. Ultimately, 12^[11,18–28]^ studies met the eligibility criteria and were included in both the qualitative and quantitative meta-analyses.

### Study and baseline characteristics

This meta-analysis included 12 studies comparing MSA and FP for the treatment of GERD. The studies, published between 2014 and 2024, comprised prospective and retrospective cohort designs with follow-up periods ranging from 30 days to over 7 years. Most studies had follow-up durations of 6 months to 3 years, with one study extending to 7 years and 4 months.

The mean age of participants ranged from 39.3 ± 12.9 to 54 ± 11.8 years for MSA and 43.8 ± 9.2 to 56.33 ± 16.31 years for FP. Sex distribution varied, with male percentages ranging from 49.6 to 88% in the MSA group and 32 to 62.5% in the FP group. Body mass index was similar across groups, with means ranging from 21.54 ± 3.59 to 29.5 ± 4.81 kg/m^2^ for MSA and 22.33 ± 3.67 to 29.7 ± 5.19 kg/m^2^ for FP. Hiatal hernia presence was reported in three studies, with sample sizes of 8–111 for MSA and 12–148 for FP. Hernia size, where reported, ranged from 1.0 ± 1.48 to 2.0 ± 1.0 cm for MSA and 1.0 ± 1.49 to 2.0 ± 1.51 cm for FP (Table 1).

**Table 1:** Detailed characteristics of each included studies and baseline characteristics

				Age (mean ± SD)		Male/female (%)		Body mass index (mean ± SD)		Hiatal hernia (*n*)	
Author	Year	Study design	Follow-up period MSA	MSA	FP	MSA	FP	MSA	FP	MSA	FP
Asti *et al*	2016	Prospective Cohort	7 years 4 months	44 ± 20	50 ± 24	32.6/67.4	59.2/40.8	23.94 ± 4.54	25.10 ± 4.47	NA	NA
Asti *et al*	2023	Prospective Cohort	12–24 months	48 ± 15.74	55.3 ± 13.63	88/42	32/37	25.17 ± 3.60	25.19 ± 3.89	NA	NA
Bonavina *et al*	2020	Prospective Cohort	3 years	46.6 ± 13.6	56.3 ± 12.6	63.7/36.3	49.4/50.6	25.7 ± 3.7	27.81 ± 4.0	NA	NA
Daus *et al*	2024	Retrospective Cohort	1 year	50.33 ± 17.19	52 ± 15.70	59/41	58/42	27.07 ± 4.19	27.33 ± 3.74	110	120
Louis *et al*	2014	Retrospective Cohort	6 months	54 ± 11.8	47 ± 12.2	52.9/47	40.6/59.3	27 ± 5.1	30 ± 4.4	NA	NA
Reynolds *et al* (a)	2015	Retrospective Cohort	1 year	53	53	61.5/38.5	46.2/53.8	26	27	35	50
Reynolds *et al* (b)	2015	Retrospective Cohort	12 months	53	54	60/40	54/26	26.4	26.7	35	35
Riegler *et al*	2014	Prospective Cohort	1 year	46.6 ± 13.9	52.8 ± 12.8	61.7/38.3	60/40	25.7 ± 3.8	26.1 ± 5.3	NA	NA
Sheu *et al*	2014	Retrospective Cohort	7 months	39.3 ± 12.9	43.8 ± 9.2	59.3/41.7	50/50	26.8 ± 4.4	26.8 ± 3.6	NA	NA
Warren *et al*	2015	Retrospective Cohort	12 months	53.33 ± 16.3	53.0 ± 15.56	52/48	43/57	28.13 ± 4	28.99 ± 4	111	148
Wisniowski *et al*	2024	Retrospective Cohort	30 days	50.33 ± 16.30	56.33 ± 16.31	49.6/50.4	34.3/65.7	29.5 ± 4.81	29.7 ± 5.19	NA	NA
Zhu *et al*	2024	Retrospective Cohort	12 months	40.19 ± 9.40	46.88 ± 16.83	50% male/50% female	62.5% male/37.5% female	21.54 ± 3.59	22.33 ± 3.67	8	12

### Clinical outcomes

**Figure 2.**
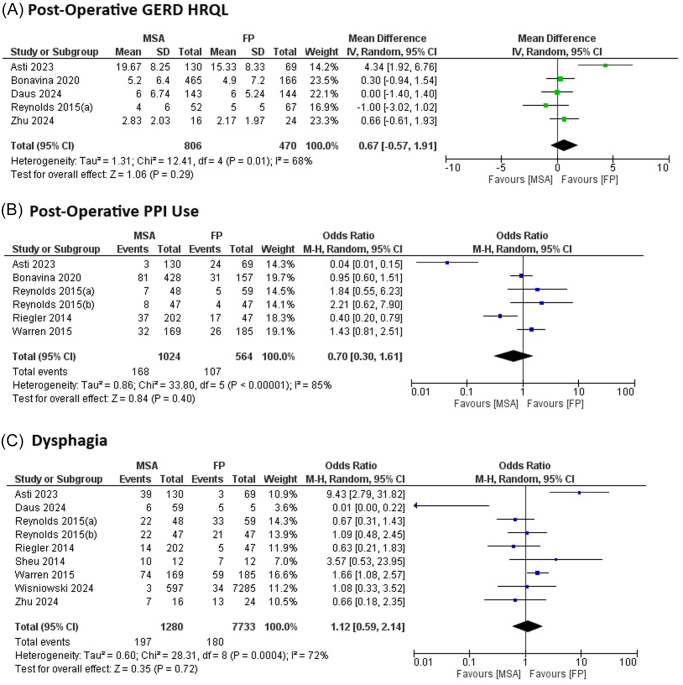
Forest plots comparing outcomes between magnetic sphincter augmentation (MSA) and fundoplication (FP) in gastroesophageal reflux disease (GERD) patients: (A) post-operative GERD HRQL, (B) post-operative proton pump inhibitor (PPI) use, and (C) dysphagia.

**Figure 3.**
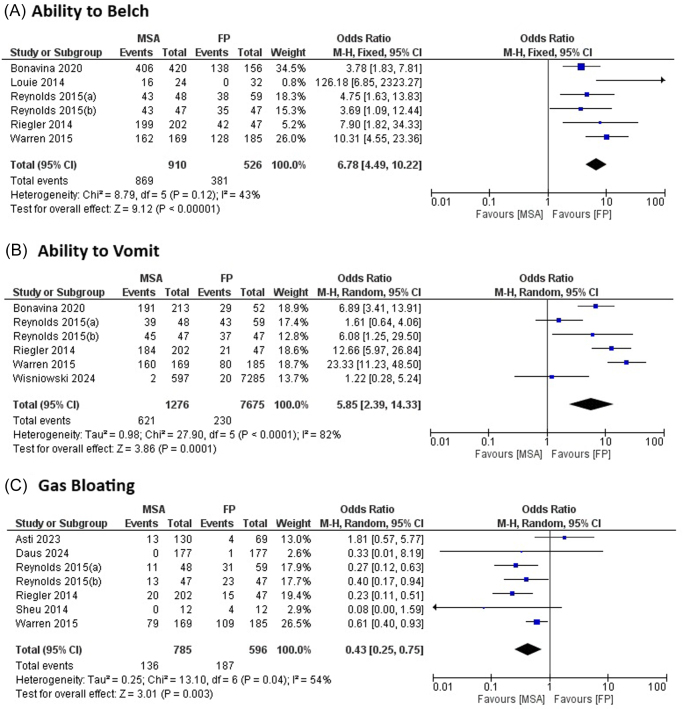
Forest plots comparing outcomes between magnetic sphincter augmentation (MSA) and fundoplication (FP) in gastroesophageal reflux disease (GERD) patients: (A) ability to belch, (B) ability to vomit, and (C) gas bloating.

- **GERD-HRQL Score**: Five studies were included in the meta-analysis comparing postoperative GERD-HRQL scores between MSA and FP. No significant difference was observed between the two groups (pooled MD = 0.67, 95% CI: –0.57–1.91, *P* = 0.29 using the random-effects model). Substantial heterogeneity was present (*I*^2^ = 68%). Sensitivity analysis, excluding Asti *et al* (2023), reduced heterogeneity to *I*^2^ = 0% (Fig. 2). This study was selected for exclusion based on leave-one-out analysis, which identified it as an influential outlier due to its high weight (contributing approximately 25% to the pooled estimate) and methodological differences, including the use of Toupet FP (partial wrap) compared to Nissen FP in most other studies, as well as its position as an asymmetry contributor in the funnel plot.
- **Postoperative PPI Use**: Six studies were included to compare postoperative PPI use between MSA and FP. No significant difference was found in the likelihood of PPI use (pooled OR = 0.70, 95% CI: 0.30–1.67, *P* = 0.40 using the random-effects model). Considerable heterogeneity was observed (*I*^2^ = 85%). Sensitivity analysis, excluding Asti *et al* (2023) and Riegler *et al* (2014), reduced heterogeneity to *I*^2^ = 0% (Fig. 2). These studies were identified through leave-one-out analysis as key contributors to heterogeneity; Asti *et al* (2023) due to its high weight and use of partial FP, and Riegler *et al* (2014) due to its multicenter observational design and potential selection bias, as indicated by its outlier position in the funnel plot and lower methodological quality score.
- **Dysphagia**: Nine studies were included, and no significant difference was observed in postoperative dysphagia between MSA and FP (pooled OR = 1.12, 95% CI: 0.59–2.14, *P* = 0.72 using the random-effects model). Substantial heterogeneity was present (*I*^2^ = 72%). Sensitivity analysis, excluding Asti *et al* (2023), reduced heterogeneity to *I*^2^ = 60% (Fig. 2). Asti *et al* (2023) was chosen for exclusion based on leave-one-out analysis, reflecting its influence on heterogeneity owing to its relatively high weight, partial FP technique, and deviation from the central cluster in the funnel plot.
- **Ability to Belch**: Six studies were included, and patients undergoing MSA demonstrated a significantly greater ability to belch compared to those undergoing FP (pooled OR = 6.78, 95% CI: 4.49–10.22, *P* < 0.00001 using the fixed-effect model). Heterogeneity was moderate (*I*^2^ = 43%; Fig. 3).
- **Ability to Vomit**: Six studies were included, and patients with MSA showed a significantly greater ability to vomit compared to those with FP (pooled OR = 5.85, 95% CI: 2.39–14.33, *P* = 0.0001 using the random-effects model). Substantial heterogeneity was present (*I*^2^ = 82%). Sensitivity analysis, excluding Reynolds *et al* (2015a), Warren *et al* (2015), and Wisniowski *et al* (2024), reduced heterogeneity to *I*^2^ = 0% (Fig. 3). These studies were selected via leave-one-out analysis as major sources of heterogeneity; Reynolds *et al* (2015a) and Warren *et al* (2015) due to their matched-pair and multi-institutional designs with potential variability in patient selection, and Wisniowski *et al* (2024) owing to its short-term (30 days) follow-up and database-derived nature, which positioned them as outliers in the funnel plot with combined weights exceeding 40%.
- **Gas Bloating**: Seven studies were included, and MSA was associated with a significantly lower risk of gas bloating compared to FP (pooled OR = 0.43, 95% CI: 0.25–0.75, *P* = 0.003 using the random-effects model). Moderate heterogeneity was observed (*I*^2^ = 54%). Sensitivity analysis, excluding Asti *et al* (2023), reduced heterogeneity to *I*^2^ = 34% (Fig. 3). Exclusion of Asti *et al* (2023) was justified by leave-one-out analysis, highlighting its impact on heterogeneity due to high weight, the partial FP approach, and its asymmetric position in the funnel plot.

### GRADE assessment

The quality of evidence for this meta-analysis was evaluated using the GRADE approach, starting at low certainty due to the observational cohort design. The risk of bias was low, with minor variations in Sheu 2014 and Louie 2014. Ability to belch (six studies) and gas bloating (seven studies) achieved moderate certainty, upgraded for large (OR = 6.78, 95% CI: 4.49–10.22, *I*^2^ = 43%) and moderate (OR = 0.43, 95% CI: 0.25–0.75, *I*^2^ = 54%) effect sizes, respectively. Dysphagia (nine studies), GERD-HRQL score (five studies), and postoperative PPI use (six studies) were rated very low certainty, downgraded for substantial heterogeneity (*I*^2^ = 72%, 68%, 85%) and imprecision (OR = 1.12, 95% CI: 0.59–2.14; MD = 0.67, 95% CI: –0.57–1.91; OR = 0.70, 95% CI: 0.30–1.67). The ability to vomit (six studies) had low certainty, downgraded for heterogeneity (*I*^2^ = 82%) but not imprecision (OR = 5.85, 95% CI: 2.39–14.33). No indirectness was noted across outcomes (Supplemental Digital Content Table S1, available at: http://links.lww.com/MS9/B67).

### Publication bias

Publication bias was assessed using funnel plots. No evidence of publication bias was observed for the ability to belch and gas bloating, with symmetrical distributions. The ability to vomit showed no strong bias, though one outlier was noted. However, evidence of publication bias was detected for dysphagia, GERD-HRQL score, and postoperative PPI use, indicated by asymmetrical plots with gaps in the lower left quadrant, suggesting potential underrepresentation of smaller studies with non-significant or negative results (Supplemental Digital Content Figure S1, available at: http://links.lww.com/MS9/B67).

### Quality assessment

The quality of the studies included was evaluated using the NOS, with scores ranging from 7 to 9 out of a possible 9. Most studies (Asti 2016, Asti 2023, Bonavina 2020, Daus 2024, Reynolds 2015, Riegler 2014, Reynolds *et al* 2015, Warren 2015, and Zhu 2024) achieved the maximum score of 9/9, indicating high quality across selection (four points), comparability (two points), and outcome (three points) domains. Louie 2014 scored 7/9, reflecting minor limitations in the outcome domain, while Wisniowski 2024 scored 8/9, with a slight shortfall in the comparability domain (Supplemental Digital Content Table S2, available at: http://links.lww.com/MS9/B67).

## Discussion

This meta-analysis provides a comprehensive comparison of MSA and FP for the management of GERD, focusing on postoperative outcomes including GERD-HRQL scores, PPI use, ability to belch and vomit, dysphagia, and gas bloating. The findings reveal no significant differences between MSA and FP in terms of GERD-HRQL scores, postoperative PPI use, or dysphagia, indicating comparable efficacy in symptom control and quality of life. However, MSA demonstrated a clear advantage in preserving physiological functions, with a significantly greater ability to belch and vomit compared to FP. Additionally, MSA was associated with a lower risk of gas bloating, a common side effect of FP. These results are supported by sensitivity analyses that reduced heterogeneity in most outcomes, enhancing the robustness of the findings. The quality of evidence, assessed using the GRADE approach, ranged from moderate to very low, reflecting the observational nature of the studies and variability in heterogeneity and precision.

The lack of significant difference in GERD-HRQL scores aligns with prior studies, such as Guidozzi *et al* (2019)^[9]^, who reported comparable quality of life improvements with MSA and FP, and Aiolfi *et al* (2018)^[7]^, whose meta-analysis found similar symptom control rates. This suggests that both procedures effectively address the core symptoms of GERD, supporting their use as viable surgical options. However, the absence of a difference in postoperative PPI use contrasts with some literature, such as Fadel *et al* (2024)^[10]^, who reported higher PPI discontinuation rates with MSA (up to 85.4%), potentially due to differences in patient selection or follow-up duration in our cohort, where heterogeneity (*I*^2^ = 85%) was a limiting factor until sensitivity analysis excluded outliers.

The superior ability to belch and vomit with MSA is consistent with its design to augment the LES while preserving natural physiology, as noted by Riegler *et al* (2015)^[11]^ and Skubleny *et al* (2017)^[8]^. These findings underscore MSA’s advantage over FP, particularly Nissen FP, which often impairs these functions due to the 360-degree wrap, as highlighted by Bonavina *et al* (2021)^[20]^. The moderate certainty of evidence for these outcomes, upgraded due to large effect sizes, strengthens the clinical relevance of this benefit.

The reduced risk of gas bloating with MSA corroborates earlier observations by Warren *et al* (2016)^[26]^, who reported lower gas-bloat syndrome rates with MSA (95.2 vs. 65.9% with FP), attributed to less disruption of gastric anatomy. This finding, supported by moderate certainty evidence, may influence patient preference and surgical decision-making, particularly for those prone to postoperative bloating^[17,23]^. Conversely, the lack of significant difference in dysphagia rates contrasts with some reports of higher transient dysphagia with MSA (e.g., Fadel *et al*, 2024)^[10]^, possibly due to our broader inclusion of studies with varying follow-up periods, which may have diluted early postoperative effects.

The substantial heterogeneity observed in several outcomes (e.g., GERD-HRQL, PPI use, ability to vomit, and dysphagia) reflects variability in study design, patient populations, and follow-up durations, a common challenge in meta-analyses of observational data. Sensitivity analyses effectively mitigated this in most cases, aligning with Higgins *et al* (2003)^[16]^, who emphasizes the importance of such approaches in managing heterogeneity. The very low certainty for GERD-HRQL, PPI use, and dysphagia, driven by imprecision and inconsistency, suggests caution in interpreting these results and highlights the need for larger, randomized controlled trials to confirm these findings.

This study integrates multi-source heterogeneous clinical data, including patient-reported outcomes and physiological function indicators, to quantify heterogeneity and optimize evidence synthesis through systematic review and meta-analysis. These efforts provide critical evidence-based support for developing AI-driven personalized surgical decision-making models in GERD management. For instance, based on patient characteristics such as age and hernia size, as well as functional needs like preserving the ability to vomit, AI systems could leverage the “efficacy-function” trade-off map derived from our findings to generate individualized surgical procedure recommendation strategies.

Recent advancements in AI have opened valuable opportunities for enhancing medical research, diagnosis, and treatment, serving as a foundation for integrating such technologies into GERD surgical decision-making. For example, the AI AlphaFold model has shown promising applications in molecular biology and drug discovery by enabling machine-learning-driven informatics investigations^[29]^. Similarly, AI models utilizing clinical images have demonstrated significant predictive value for genomic prediction and tailored treatment in cancer patients^[30]^. These innovations highlight AI’s capacity to process complex, multi-dimensional data and uncover patterns that inform personalized care.

Building on these AI advances, our meta-analysis reveals important insights for the intelligent transformation of GERD management. Current medical research is shifting from traditional evidence-based decision-making to data-driven intelligent paradigms. The core value of this study lies in systematically deconstructing the complex trade-off between surgical efficacy and functional preservation in GERD, a key challenge in personalized medicine. This involves transforming multi-dimensional heterogeneous clinical evidence, such as patient-reported outcomes, anatomical features, and functional requirements, into quantifiable decision-making frameworks. AI technologies, particularly deep learning and knowledge graphs, excel at integrating massive medical data and revealing implicit diagnostic and therapeutic patterns, such as analyzing radiomics features for treatment response prediction or constructing dynamic clinical pathways from real-world data.

The “efficacy-functionality” paradox identified here, exemplified by MSA’s advantages in preserving physiological functions and its indication boundaries, offers a structured knowledge framework for AI models. Future research can build on this evidence-based foundation by incorporating multimodal real-time data, including intraoperative dynamic physiological monitoring and postoperative long-term electronic health records to train intelligent systems. These systems could dynamically optimize surgical procedure selection, facilitating a transition from population-level evidence to individual-level optimal solutions. This extension not only imbues traditional meta-analyses like ours with contemporary significance but also charts a key evolutionary direction for intelligent decision-making in GERD surgery.

Limitations of this meta-analysis include the reliance on observational studies, which, despite high NOS scores, lack the rigor of randomized trials, potentially introducing selection bias. The exclusion of non-English studies and pediatric populations may limit generalizability, while the variability in follow-up periods (30 days to 7+ years) could affect outcome consistency. Additionally, the paucity of data on long-term complications warrants further investigation.

## Conclusion

In conclusion, this meta-analysis supports MSA as a promising alternative to FP for GERD, particularly for patients valuing preserved physiological functions and reduced gas bloating. The comparable efficacy in symptom control and quality of life with FP suggests that both procedures remain viable, with choice depending on patient-specific factors such as age, comorbidities, and anatomical considerations (e.g., hiatal hernia size). Future research should focus on randomized trials with standardized follow-up to address heterogeneity and enhance the certainty of evidence, while also exploring AI-integrated approaches to refine personalized surgical strategies for GERD management, as outlined in the discussion above.

## Data Availability

No new data generated.

## Research registration unique identifying number (UIN)

CRD42024564433.
